# Cisplatin Resistance and Metabolism: Simplification of Complexity

**DOI:** 10.3390/cancers16173082

**Published:** 2024-09-04

**Authors:** Nikolay V. Pervushin, Maria A. Yapryntseva, Mikhail A. Panteleev, Boris Zhivotovsky, Gelina S. Kopeina

**Affiliations:** 1 Faculty of Medicine, Lomonosov Moscow State University, 119991 Moscow, Russia; rhododendron.nick@mail.ru (N.V.P.); smariaal@mail.ru (M.A.Y.); 2 Engelhardt Institute of Molecular Biology, Russian Academy of Sciences, 119991 Moscow, Russia; 3 Department of Medical Physics, Physics Faculty, Lomonosov Moscow State University, 119991 Moscow, Russia; mikhail.panteleev@fccho-moscow.ru; 4 Dmitry Rogachev National Medical Research Center of Pediatric Hematology, Oncology and Immunology, Ministry of Healthcare of Russian Federation, 117198 Moscow, Russia; 5 Center for Theoretical Problems of Physicochemical Pharmacology, Russian Academy of Sciences, 109029 Moscow, Russia; 6 Division of Toxicology, Institute of Environmental Medicine, Karolinska Institutet, P.O. Box 210, 17177 Stockholm, Sweden

**Keywords:** cisplatin, resistance, metabolism, apoptosis, cancer

## Abstract

**Simple Summary:**

Prolonged cisplatin treatment can lead to acquired cancer cell resistance accompanied by various metabolic disturbances, which play an important role in maintaining homeostasis. In particular, resistant cells can regulate energy requirements and evade cisplatin-mediated toxicity via multiple metabolic adaptations. Here we discuss the existing evidence on metabolic alterations in cisplatin-resistant cells and their impact on the development of drug non-response.

**Abstract:**

Cisplatin is one of the most well-known anti-cancer drugs and has demonstrated efficacy against numerous tumor types for many decades. However, a key challenge with cisplatin, as with any chemotherapeutic agent, is the development of resistance with a resultant loss of efficacy. This resistance is often associated with metabolic alterations that allow insensitive cells to divide and survive under treatment. These adaptations could vary greatly among different tumor types and may seem questionable and incomprehensible at first glance. Here we discuss the disturbances in glucose, lipid, and amino acid metabolism in cisplatin-resistant cells as well as the roles of ferroptosis and autophagy in acquiring this type of drug intolerance.

## 1. Introduction

Cancer drug resistance is a serious challenge in the therapy of numerous tumors, often leading to treatment failure and a poor prognosis [1]. Platinum drugs, such as oxaliplatin, carboplatin, and especially cisplatin, are commonly used to treat cancer. These potent alkylating chemotherapeutics can form covalent bonds to DNA via purine bases, causing DNA interstrand cross-links. Platinum-induced stress inhibits DNA replication, arrests the cell cycle, and activates programmed cell death (PCD), including apoptosis [2].

**Figure 1:**
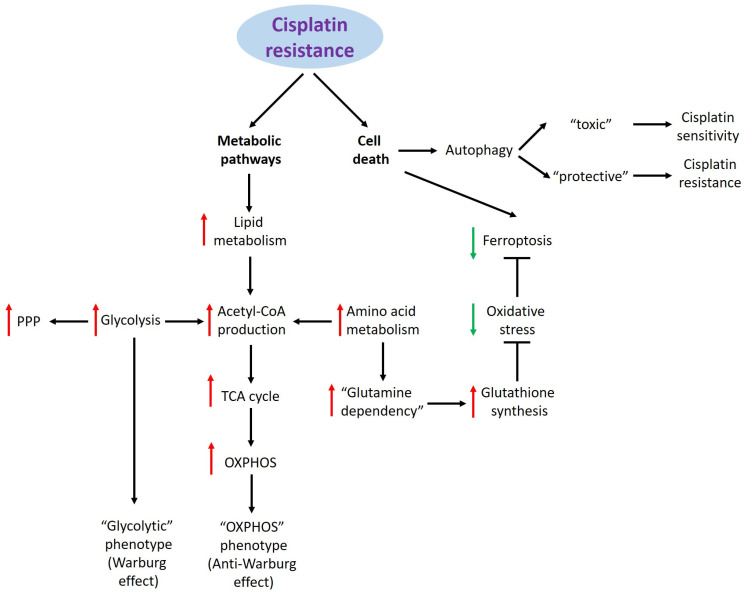
Alterations in metabolism and programmed cell death regulation of cisplatin-resistant cancer cells. Notes: red and green arrows depict an increase and a decrease of activity of selected processes; PPP—pentose phosphate pathway; Acetyl-CoA—acetyl coenzyme A; TCA—tricarboxylic acid; OXPHOS—oxidative phosphorylation.

Cisplatin was first synthesized in the mid-nineteenth century. More than 100 years later, in 1978, it was approved for clinical application. Cisplatin and its derivatives have been found to possess high anti-cancer activity both in the form of monotherapy and when combined with other medications for various solid and hematological malignancies such as leukemia, lymphomas, breast, testicular, ovarian, head and neck, cervical, sarcomas, and other cancers [3,4]. However, the efficacy of platinum drugs can become limited with the development of acquired drug tolerance [5]. Thousands of articles focusing on cisplatin resistance have been published to date, and the mechanisms underlying non-response to platinum drugs have been reviewed in detail [6,7,8,9,10,11,12]. Briefly, the main causes of cancer cell insensitivity to cisplatin include drug-reduced uptake/increased efflux, glutathione-mediated inactivation, mutations in oncosuppressor genes, DNA damage repair, epigenetic regulation, and others that lead to apoptosis evasion [8]. As a result, cisplatin-resistant (CR) cells are characterized by specific features, particularly avoidance of cell death and modulation of proliferative and metabolic activities. These processes are tightly regulated and permit CR cells to retain viability in stressful conditions (Figure 1). Importantly, cisplatin resistance promotes suppression of not only apoptosis but also other forms of cell death, including ferroptosis and autophagy.

Metabolic reprogramming is one of the key hallmarks of cancer [13]. Rapid and uncontrolled proliferation of tumor cells is an energy-consuming process, and high bioenergetic capacity is necessary to maintain it [11]. CR cells are characterized by an altered metabolism that contributes to drug intolerance [12]. These metabolic disturbances could vary among different tumor types, leading to misunderstanding of their underlying mechanisms. Our commentary aims to elucidate several controversial issues in this regard. For instance, CR cells often demonstrate exacerbated glycolytic phenotype, but in several cases, they may also undergo reverse shift [14]. Moreover, the levels of different metabolic-related proteins such as glucose transporters [14], monocarboxylate transporters [14,15], and fatty acid synthase [9,16] can be altered in opposite directions in various models of cisplatin resistance.

## 2. Cisplatin Resistance and Metabolic Pathways

### 2.1. Glucose Metabolism

Glucose is the primary energy source for cells. Briefly, glucose metabolism includes five steps: uptake by glucose transporters (GLUTs); conversion into pyruvate during glycolysis; generation of acetyl coenzyme A (acetyl-CoA) from pyruvate; activation of the tricarboxylic acid (TCA) cycle, producing reduced Nicotinamide adenine dinucleotide (NADH) and Flavin Adenine Dinucleotide (FADH2); and oxidative phosphorylation (OXPHOS), which involves NADH, FADH2, the electron transport chain, and Adenosine triphosphate (ATP) synthesis via ATP synthase. The second and third steps occur in the cytoplasm, and the latter takes place in the mitochondria. Under normal oxygen conditions, classical glucose catabolism is well-understood [17]. However, in hypoxia, OXPHOS is unavailable, and pyruvate is catalyzed to lactate by lactate dehydrogenase, resulting in rapid ATP production. Lactate is the final product of anaerobic glucose metabolism and is exported out of cells [12,17]. The pentose phosphate pathway (PPP), another essential branch of glycolysis, is required for nucleotide synthesis and reactive oxygen species (ROS) detoxification [18].

Cancer cells need large amounts of energy and biosynthetic materials to sustain their high proliferation rates. Accordingly, they adapt glucose metabolism to meet these demands. The most well-known adaptation is a phenomenon called the Warburg effect, which represents a shift from OXPHOS to glycolysis even in aerobic conditions [19]. This alteration is supported by high glucose consumption, achieved by increased gene expression of Glucose transporter (GLUTs), especially *GLUT1* and *GLUT3*. This increased expression can be induced by several oncogenes, including *EGFR*, *RAS*, *MYC*, and others [20].

Glucose uptake and glycolysis enhancement are also associated with cisplatin resistance. Under some conditions, CR cells may exhibit a glycolytic phenotype characterized by upregulation of GLUT1, glycolytic enzymes (e.g., hexokinase 2, enolase 1), inhibitors of acetyl-CoA production (pyruvate dehydrogenase kinases), or monocarboxylate transporters, which export lactate out of the cells to prevent suppression of glycolysis (Table 1). Gene knockdown or pharmacological inhibition of these targets was found to restore cisplatin sensitivity [10,11,12].

In a recent publication by Afonso et al. [14], three urothelial bladder cancer (UBC) cell lines were adapted to cisplatin treatment, resistant sublines were produced, and their metabolic profiles were analyzed using multiple approaches. The cell line with the highest resistance rate (HT1376) demonstrated enhanced glucose uptake and GLUT3 expression and a higher basal glycolysis level. Conversely, the GLUT1 level was decreased in CR cells, probably due to redundancy with GLUT3. Accordingly, the Warburg effect in this cell line was exacerbated, providing a resistance mechanism [14].

The preference of cancer cells for glycolysis over OXPHOS could be explained by faster ATP production. Furthermore, a significant change in cancer glycolytic activity is associated with the activation of the PPP, which supports a high proliferation rate by enhancing nucleotide synthesis. Activation of aerobic glycolysis and the subsequent secretion of lactate from cells result in the diversion of a pool of carbon from biosynthetic pathways, which does not align with the metabolic demands of proliferating cancer cells. It has been hypothesized that the glycolytic phenotype maintains high levels of glycolysis intermediates, promoting the PPP as a biosynthetic branch of glycolysis [26]. Additionally, the PPP counteracts oxidative stress, enhancing cancer cell viability and drug resistance. Inhibition of several PPP enzymes (e.g., glucose-6-phosphate dehydrogenase, transketolase), which are elevated in CR cells of various origins, was revealed to overcome cisplatin resistance [27].

The role of mitochondrial respiration in cisplatin resistance remains unclear. Almost a century ago, Otto Warburg stated that “the respiration of all cancer cells is damaged”, meaning that defects in the mitochondrial electron transport chain are essential for tumor development. Indeed, many oncogenic mutations target proteins involved in respiration [28]. Nevertheless, although mitochondrial respiration is impaired in tumor cells relative to the high rate of glucose uptake, favoring glycolysis, these cells still consume oxygen. Evidence suggests that functional mitochondria are essential for cancer cells both in culture and in humans, and OXPHOS is not compromised in these contexts [29,30,31]. The glycolytic shift described earlier is sustained by the upregulation of glycolytic enzymes in addition to high mitochondrial activity, rather than replacing it [32].

In some cases, however, CR cells demonstrate a decrease in OXPHOS. Thus, in the above-mentioned publication [14], mitochondrial activity, respiration, and ATP production were shown to be decreased in CR HT1376 cells, consistent with a more prominent glycolytic phenotype. Interestingly, another UBC cell line in this study (KU1919) showed reversed alterations in glucose metabolism (i.e., low glucose uptake and high OXPHOS levels), reverting to a preference for oxygen consumption. This metabolic re-reprogramming is also called the second metabolic switch or anti-Warburg effect, in contrast to the traditional glycolytic shift. CR cells can be also characterized by reduced levels of GLUTs and glycolytic enzymes along with heightened respiration [33]. Lower reliance on glucose consumption forces cells to use new sources of carbon, such as glutamine (discussed below). Similar metabolic remodeling has been discovered in circulating tumor cells isolated from melanoma, lung cancer, prostate cancer, and breast cancer by single-cell transcriptomic analyses [34]. Furthermore, this respiratory phenotype of epithelial cancer cells can be supported by metabolically reprogrammed stromal cells. After these cells transform into cancer-associated fibroblasts, they undergo aerobic glycolysis and secrete lactate and pyruvate, which are used by cancer cells as energy-rich substrates for the TCA cycle and subsequent OXPHOS [35]. Interestingly, resistant UBC cells exhibit lower levels of monocarboxylate transporter 4 (a lactate transporter) and extracellular lactate, indicating extensive lactate import [14]. However, mitochondrial activity was found to be dormant in this cell line because the study was conducted using a two-dimensional culture, which does not allow investigation of possible heterogeneity in the metabolic profiles of these cells; therefore, it was not possible to assert the involvement of the anti-Warburg effect [14]. Another possible explanation for high lactate uptake is glucose depletion due to active uptake and the need for other substrates. Notably, similar to GLUTs, the differences in MCT rates in various models may be linked with function redundancy, which is inherent to closely related proteins from one family. Thus, these members could replace each other in a cell- and tissue-dependent manner.

In addition to their role in energy production, mitochondria are the primary source of ROS and are essential in managing ROS levels within cells [36]. Cancer cells have elevated ROS levels, which amplify the tumorigenic phenotype, promote genomic instability, sustain proliferation, and potentially help avoid cell death. Thus, oxygen consumption by cancer cells seems to exert regulatory functions rather than compensate for dysfunctional OXPHOS [37]. Nevertheless, excessive ROS generation can induce cell death via different mechanisms, including apoptosis and mitochondrial permeability transition pore-mediated necrosis [38]. Additionally, many tumors develop a hypoxic microenvironment due to their rapid growth, which outpaces their vascular supply. Hypoxic conditions promote enhanced production of superoxide radicals within mitochondria, representing an important mechanism of tumor metabolic reprogramming, proliferation, and survival [39].

ROS accumulation and subsequent damage to DNA and other biopolymers are also part of the mechanisms underlying cisplatin cytotoxicity [40]. Notably, CR cells can modulate ROS levels. For instance, one study showed that these cells had lower basal levels of ROS, which did not increase upon cisplatin treatment compared with parental cells in UBC cell lines [41]. Nevertheless, CR cells, reverting to OXPHOS instead of glycolysis, demonstrated both higher electron leakage from the electron transport chain and higher basal levels of ROS [9,33,42]. Accordingly, elevated ROS levels were observed in all CR UBC cells [14]. Interestingly, this effect occurred independently from respiration activation, indicating a complex link between cisplatin resistance and glucose metabolism remodeling in a tissue- and context-dependent manner.

Other research has also shown that CR cells demonstrate high glycolysis and OXPHOS activity and the ability to switch between these processes, whereas chemosensitive cells rely preferentially on glycolysis in established and patient-derived ovarian cancer cell lines. High metabolic activity seems to support the energy demands of proliferation, while flexibility in metabolic pathway selection represents cellular adaptability. Low-dose cisplatin exposure leads to enhanced metabolic activity and OXPHOS responses [43]. Activation of OXPHOS metabolism and independence from glycolysis have been confirmed as mechanisms of cisplatin resistance [44,45,46]. These processes represent an escape from the action of cisplatin through accelerated proliferation and metabolic adaptation to increased energy needs. Nevertheless, the action of cisplatin is based on both cytotoxic and cytostatic effects; therefore, resistance mechanisms can vary. In our recent study, two distinct mechanisms of resistance were observed in four isogenic pairs of sensitive and CR cells originating from various tumor types. In one case, intolerance was based on the evasion of cell death with no significant alteration of energy metabolism. The other cells developed cisplatin resistance via a cytostatic mechanism, i.e., they showed decreases in their proliferation rate and metabolic (glycolytic and respiratory) activity. Because cisplatin mainly kills rapidly dividing cells, such a phenotype may increase cell survival during prolonged treatment [47].

### 2.2. Lipid Metabolism

Lipids are complex and heterogeneous groups of molecules including fatty acids, glycerides, steroids, sphingolipids, and lipoproteins. Their metabolism refers to lipid uptake, de novo synthesis, transport, and degradation, and it is essential for the maintenance of cellular structures, energy supply, and signal transduction. Particularly, in cancer cells, lipid metabolism is often upregulated for cell membrane production and energy supply through the β-oxidation of fatty acids [48]. The particularities of the molecular mechanisms of lipids conferring resistance to cisplatin were discussed in detail elsewhere [12].

Afonso et al. [14] found that enhanced lipid metabolism was also present in CR HT1376 cells. In particular, the levels of enzymes involved in fatty acid synthesis, such as acetyl-CoA carboxylase and fatty acid synthase, were higher in CR cells than in parental cells [14]. Notably, lipids, which serve as important energy sources, structural elements, and signaling molecules, are also involved in the development of cisplatin resistance [12,49]. Various stages of lipid metabolism, including phospholipid synthesis, acetyl-CoA production, and fatty acid synthesis, may be enhanced in CR cells via the upregulation of specific enzymes (Table 2). Suppression of these enzymes, such as with fatty acid synthase inhibitors (e.g., C75 or orlistat) [16,50,51], has been found to increase cisplatin sensitivity and inhibit cell proliferation. Hence, these molecules may serve as predictive biomarkers of non-response to cisplatin as well as potential drug targets. Interestingly, the fatty acid synthase levels could be increased or decreased in various CR cells (Table 2). These differences might be caused by their varied dependency on fatty acid synthesis. Additionally, the cell membrane structure may become altered [12] and less permeable in breast [52] and lung [53] CR cancer cells, thereby impairing drug transport into tumor tissues.

### 2.3. Amino Acid Metabolism

CR cells are also characterized by dysregulated amino acid metabolism, which allows them to survive and proliferate under stressful conditions. Amino acids are mainly involved in dissimilation/biosynthesis processes and the maintenance of redox homeostasis in CR cells. Altered levels of several amino acids (specifically, decreased rates of alanine and tyrosine) and increased rates of glutamate have been detected [14]. The latter change is in agreement with other reports: cisplatin resistance is associated with glutamine dependency. For instance, CR cells have been observed to be more susceptible to platinum drugs upon glutamine deprivation in lung and ovarian tumors [56,57]. Additionally, the levels of several enzymes responsible for glutamine utilization as a source of energy via the TCA cycle and subsequent OXPHOS (e.g., glutaminase [57] and glutamate oxaloacetate transaminase 1 [58]) are elevated in CR cells [12]. Notably, CR cells reportedly become less dependent on the thioredoxin antioxidant system [33,59], which is targeted by cisplatin [60]. Hence, high glutamine consumption, which is essential for increased glutathione synthesis, underlies the ability of CR cells to remain viable under cisplatin-induced genotoxic stress [33]. This circumstance may explain the enhanced expression of glutamine transporters (ASCT2/SLC1A5) in CR cells [12].

Besides glutamine, other amino acids may contribute to cisplatin resistance (Table 3). CR cells consume high levels of tryptophan, which is metabolized via the kynurenine pathway to synthesize increased levels of NAD+. This further contributes to energy production and ROS detoxification [61]. However, CR cells are also able to survive under conditions of low NAD+ [33]. Inhibition of high methionine consumption was recently found to resensitize bladder CR cells to cisplatin [62]. Additionally, deprivation of not only glutamine or methionine but also arginine may lead to restoration of cisplatin sensitivity [33]. CR cells were shown to exhibit epigenetic silencing of Argininosuccinate Synthase 1 (ASS1), a key enzyme of the urea cycle, and become more arginine-dependent [63]. Specific inhibition of amino acid biomarkers of cisplatin resistance (e.g., glutaminase suppression by telaglenastat/CB-839 or Bis-2- (5-phenylacetamido-1,3,4-thiadiazol-2-yl)ethyl sulfide (BPTES) [10,11], glutamine channel blockade by sulfasalazine or erastin [64], and indoleamine 2,3-dioxygenase-1 (IDO1) inhibition by Epacadostat [10,11]) may enhance the efficacy of chemotherapeutics and overcome this type of drug insensitivity.

Finally, numerous proteins may also be considered as predictors of cisplatin resistance. For instance, metallothioneins, which are cysteine-rich proteins, participate in the regulation of redox homeostasis and detoxification. Similar to glutathione, enhanced expression of these low-molecular-weight proteins is associated with the development of acquired cisplatin non-response [8,65]. Moreover, low levels of members of the heat-shock protein family (e.g., HSP70 and HSP90), which modulate cell protection under stressful conditions, are associated with low cisplatin sensitivity in ovarian cancer [66,67]. Hence, targeting these proteins could be a potential therapeutic strategy against cisplatin resistance.

## 3. Cisplatin Resistance and Cell Death

The most studied cell death pathway induced by cisplatin is apoptosis. There is plenty of research devoted to this topic [2,3,68]. To avoid overlapping, we decided to concentrate on less understood metabolism-related modes of cell death.

### 3.1. Ferroptosis

Platinum drugs can induce not only apoptosis but also other types of PCD [2], including ferroptosis, which has been actively studied in the past decade [69,70]. Ferroptosis is associated with iron accumulation and lipid peroxidation. Although it has specific morphological and biochemical properties similar to those of other types of PCD, ferroptosis is actually considered a type of regulated necrotic-like death [71]. The cystine/glutamate antiporter SLC7A11 (also known as xCT) (SLC7A11/xCT) and glutathione peroxidase 4 play a crucial role in ferroptosis regulation. SLC7A11/xCT is a specific transport channel that participates in extracellular cystine uptake in exchange for glutamate. Cystine reduction to cysteine is necessary for the synthesis of glutathione, which rescues cells from oxidative stress [72]. Glutathione peroxidase 4 is an enzyme that removes lipid peroxides and suppresses ferroptosis activation. As ferroptosis enhances the cytotoxic action of cisplatin, this enzyme counteracts it [8,71]. Numerous studies have recently demonstrated that this type of PCD contributes to cisplatin non-response. Generally, ferroptosis inhibition or resistance promotes the development of intolerance to cisplatin, whereas insensitivity to platinum drugs results in the suppression of ferroptosis in various tumor models [73,74]. Overall, induction of ferroptosis via several signaling pathways (examples are presented in Table 4) may be a prospective strategy for alleviating cisplatin resistance.

### 3.2. Autophagy

The other cisplatin-induced type of PCD is autophagy, which displays dual roles in oncogenesis depending on the cellular context [2]. Generally, autophagy promotes the removal of damaged molecules and organelles [82]. Thus, excessive autophagic processes may cause cell death and enhance the cytotoxicity of cisplatin (i.e., cytotoxic autophagy). As an example, disturbed autophagy can result in decreased degradation and therefore stabilization of some pro-survival factors such as long non-coding RNAs, which promote chemoresistance [83]. In most cases, however, autophagy serves as a survival mechanism, supporting the nutrition and energy demands of cancer cells exposed to various stressful stimuli and promoting cisplatin resistance (i.e., cytoprotective autophagy) [8,82]. Importantly, the basal level of autophagy is significantly elevated in CR cells [84]. In particular, an elevated hexokinase 2 level has been found to activate cisplatin-mediated autophagy, which protects tumor cells [22]. Furthermore, mitophagy (a specific form of autophagy) is responsible for the utilization of damaged mitochondria [85], thereby maintaining mitochondrial homeostasis and supporting tumor proliferation, progression, and drug resistance [86,87]. Numerous reports indicate that autophagy inhibition might therefore have clinical significance in cancer treatment [84,88].

Nevertheless, it should be noted that cisplatin can induce both toxic and protective modes of autophagy [8]. Interestingly, cisplatin may also activate the non-protective subtype of autophagy when autophagy suppression does not significantly influence sensitivity to treatment. The switch between protective and non-protective autophagy modes was shown to be p53-dependent in non-small cell lung cancer cells [89,90], but the significance of non-protective autophagy in anticancer therapy is incompletely understood. Thus, autophagy commonly acts as a cytoprotective mechanism that promotes cisplatin resistance. However, the presence of other types of autophagy complicates the possible use of autophagy inhibitors to overcome drug resistance in clinics [84]. The effectiveness of this approach remains uncertain and requires further research.

## 4. Conclusions

Metabolic alterations are of great importance for the acquired resistance of cancer cells to cisplatin. They also may be associated with different cisplatin-mediated types of PCD. Notably, CR cells are able to suppress ferroptosis and activate cytoprotective autophagy, which is essential to maintain viability under drug exposure. The interplays between metabolic changes and various PCD pathways play a critical role in the development of cisplatin intolerance (Figure 1). However, there are still several questions concerning the metabolism of CR cells. First, can these disturbances serve as prognostic markers of malignancy? The answer is likely “yes” because many changes in glucose, lipid, and amino acid metabolism are associated with poor patient outcomes [12]. Second, can these biomarkers serve as drug targets? Again, the answer is likely “yes”. Several studies have demonstrated that inhibition of specific participants in metabolic pathways can enhance cisplatin-induced cell death and overcome resistance to this drug [8,11,12]. Third, and perhaps most important, can these therapeutic approaches be effective in humans? The answer remains controversial and unclear. Much research in this field is conducted on two-dimensional cell culture models which do not represent the interaction of different cell types in tumor tissue. Also, many associations between metabolic changes and resistant phenotype were revealed whereas their mechanisms remain unclear. Well-defined preclinical models are needed for the discovery of new metabolic-related biomarkers and the development of new target drugs. Some new approaches were recently reviewed [91,92]. Failures in clinical trials are usually associated with significant toxic effects on healthy tissues and the low efficacy of experimental drugs. The latter may be explained by the complexity of metabolic alterations in CR cells, as discussed above. CR cells are able to utilize various metabolic pathways to support their survival in toxic conditions. High metabolic activity supports their proliferation; therefore, resistant cells often enhance their glycolytic machinery to meet high energy and biosynthesis demands. Some of them exhibit a reverse shift back to OXPHOS metabolism, which is accompanied by lower glucose dependency. Moreover, resistant cells can develop a metabolic phenotype characterized by low activity and slow proliferation. In such cases, the growth of tumor cells is suppressed, allowing CR cells to escape death. Another issue to be considered is the timing of metabolic changes during cisplatin treatment. Cisplatin action includes its uptake into cells, DNA binding, DNA damage response signaling, induction and development of cell death, and excretion from organisms. The open question is when metabolism-targeting drugs more potently sensitize cancer cells to cisplatin.

Notably, tumor cells are prone to using multiple compensatory mechanisms to maintain their viability. CR cells are no exception, and this may be illustrated by an example of ROS protection. As mentioned above, cisplatin has been found to impair the thioredoxin antioxidant system, and CR cells could compensate for this by elevated glutathione synthesis. It is logical to assume that a long-term combination strategy using platinum drugs and inhibitors of glutamine metabolism might increase the activation of other protection systems, such as the PPP, the kynurenine pathway, or augmented metallothioneins synthesis, all of which are also involved in the modulation of redox homeostasis. Taken together, numerous reports indicate that the mechanisms underlying cisplatin resistance seem to be context-dependent and determined by multiple factors in various cancer types. Further investigations may elucidate these mechanisms and make them more comprehensible and predictable in the future.

## Figures and Tables

**Table 1:** Potential predictive markers of cisplatin resistance associated with glucose metabolism.

Biomarkers	Functions	Rate in (CR) Cisplatin-Resistant Cells	Tumor Models (References)
GLUTs	Glucose uptake	Controversial (GLUT1—Increased [21]/Decreased [14]; GLUT3—Increased [14])	Head and neck squamous cell carcinoma (HNSCC) [21] Urothelial bladder cancer [14]
Hexokinase 2 (HK2)	Glucose-6-phosphate generation (1 step of glycolysis)	Increased	Ovarian cancer [22]
Enolase 1 (ENO1)	Phosphoenolpyruvate (9 step of glycolysis)	Increased	Gastric cancer [23]
Pyruvate dehydrogenase kinase 1 (PDK1)	Pyruvate dehydrogenase inhibition	Increased	Ovarian cancer [24]
Pyruvate dehydrogenase kinase 2 (PDK2)			Head and neck cancer [25]
Monocarboxylate transporters (MCTs)	Lactate acid efflux	Controversial (MCT1—Increased [15]; MCT4—Decreased [14])	Ovarian cancer [15] Urothelial bladder cancer [14]

**Table 2:** Potential predictive markers of cisplatin resistance associated with lipid metabolism.

Biomarkers	Functions	Rate in Cisplatin-Resistant (CR) Cells	Tumor Models (References)
Alkylglyceronephosphate synthase (AGPS)	Phospholipid synthesis	Increased	Glioma [54]
Acyl-coenzyme A synthetase 2 (ACSS2)	Acetyl-CoA production	Increased	Bladder cancer [55]
Acetyl-CoA-carboxylase (ACC)	Fatty acid synthesis	Increased	Lung cancer [9]
Fatty acid synthase (FAS)	Fatty acid synthesis	Controversial (Increased [9]/Decreased [16])	Lung cancer [9] Ovarian cancer [16]

**Table 3:** Potential predictive markers of cisplatin resistance associated with amino acid metabolism.

Biomarkers	Functions	Rate in Cisplatin-Resistant (CR) Cells	Tumor Models (References)
Alanine-serine-cysteine transporter 2 (ASCT2/SLC1A5)	Glutamine transport	Increased	Ovarian cancer [57]
Glutaminase (GLS)	Glutamine hydrolysis to glutamate	Increased	Ovarian cancer [57]
Glutamate oxaloacetate transaminase 1 (GOT1)	Oxaloacetate generation	Increased	Various cancer models [58]
Glutamate dehydrogenase (GLUD1)	α-ketoglutarate generation	Decreased	Various cancer models [58]
Indoleamine 2,3-dioxygenase-1 (IDO1)	Tryptophan utilization	Increased	Lung cancer [61]
Argininosuccinate synthetase (ASS1)	Arginine synthesis	Decreased	Ovarian cancer [63]
Methionine adenosyl transferase IIa (MAT2A)	S-adenosylmethionine (SAM) generation	Increased	Bladder cancer [62]

**Table 4:** Associations between ferroptosis and cisplatin resistance.

Biomarkers	Signaling Pathway	Functions	Ferroptosis Regulation	Tumor Models (References)
Glutathione peroxidase 4 (GPX4)	STAT3/Nrf2 (transcriptional activation)	Lipid peroxidation protection	Inhibition	Osteosarcoma [75] Non-small cell lung cancer (NSCLC) [76]
Cystine/glutamate antiporter SLC7A11/xCT		Cystine transport	Inhibition	Head and neck cancer [77]
	HMGA1/ATF4 (transcriptional activation)			Esophageal squamous cell carcinoma (ESCC) [78]
	FAM120A (translational activation)			Gastric cancer [79]
	SPTBN2 (posttranslational activation)			NSCLC [80]
Glutamate–cysteine ligase (GCLC)	PAX8 (transcriptional activation)	Glutathione synthesis	Inhibition	Ovarian cancer [81]

## Data Availability

Data are contained within the article.
